# Supplementary material for: Listeria adhesion protein orchestrates caveolae-mediated apical junctional remodeling of epithelial barrier for Listeria monocytogenes translocation
Source: mBio. 2024 Feb 20;15(3):e02821-23. doi: 10.1128/mbio.02821-23 (PMC10936185; doi:10.1128/mbio.02821-23)
Supplement: Supplemental material — Supplemental tables and figures. [file mbio.02821-23-s0001.pdf]

Supplemental Tables and Figures

Listeria adhesion protein orchestrates caveolae-mediated apical junctional remodeling of epithelial barrier for *L. monocytogenes* translocation

Rishi Drolia*[1,2,3,4], Donald B. Bryant[3], Shivendra Tenguria[1,4], Zuri A. Jules-Culver[2] , , Jessie Thind[3], Breanna Amelunke[3], Dongqi Liu[1,4], Nicholas L.F. Gallina[1,4], Krishna K. Mishra[1], Manalee Samaddar[1,4], Manoj R. Sawale[1], Dharmendra K. Mishra[1], Abigail Cox[5], & Arun K. Bhunia*[,1,4,5]

[1]Molecular Food Microbiology Laboratory, Department of Food Science, Purdue University, West Lafayette, Indiana, USA
[2]Department of Biological Science, Old Dominion University, Norfolk, Virginia, USA
[3]Department of Biological Science, Eastern Kentucky University, Richmond, Kentucky, USA
[4]Purdue Institute of Inflammation, Immunology, and Infectious Disease, Purdue University, West Lafayette, Indiana, USA
[5]Department of Comparative Pathobiology, Purdue University, West Lafayette, Indiana, USA

***Correspondence:** bhunia@purdue.edu; rdrolia@odu.edu

TABLE S1. Bacterial strains used in the study

Bacterial Strains	Background	Source (Reference)
L. monocytogenes F4244 (WT), serovar 4b, a clinical isolate		CDC, Atlanta, GA (1)
L. monocytogenes KB208 (*lap*⁻)	F4244 (WT)	Our Lab (2)
L. monocytogenes AKB301 (Δ*inlA*)	F4244 (WT)	Our Lab (3)
L. monocytogenes CKB (*lap*⁻*lap*⁺)	F4244 (WT)	Our Lab (2)
L. monocytogenes (Δ*inlAlap*⁻)	F4244 (WT)	This Study
L. monocytogenes F4244 (InlA^{m})	F4244 (WT)	Our Lab (4)
L. monocytogenes (InlA^{m}*lap*⁻)	InlA^{m}	This Study
L. monocytogenes F4244 (Em^{R})	F4244 (WT)	Our Lab (2)
L. innocua F4248 (WT)		Our Lab

TABLE S2. Chemicals, antibodies, reagents and software used

Items	Source (Ref)	Catalog #
**Antibodies**		
Rat anti-microfold (M) cell antibody	Miltenyi Biotec	Cat # 130-096-148, RRID: AB_2660297
Rat anti-ZO-1 Antibody	Thermo Fisher Scientific	Cat # MABT11MI, RRID: AB_628459
Rabbit polyclonal anti-*Listeria*	Our Lab (5)	N/A
Rabbit polyclonal anti-InlA	Our Lab	N/A
Mouse monoclonal anti-LAP	Our Lab	N/A
Mouse monoclonal ant-InlA	Mendonca et al (6)	N/A
Horse anti-mouse IgG (HRP-linked)	Cell Signaling	Cat # 7076, RRID: AB_330924
Goat anti-rabbit IgG (HRP-linked)	Cell Signaling	Cat # 7074, RRID: AB_2099233
Rabbit polyclonal anti-Muc-2	Novus Biological	Cat # NBP1-31231, RRID: AB_10003763
Rabbit polyclonal anti-Cleaved-caspase 3 (Asp175)	Cell Signaling	Cat # 9661S, RRID: AB_2341188
Mouse monoclonal anti-MLCK	Sigma-Aldrich	Cat # M7905, RRID: AB_477243
Rabbit polyclonal phosphorylated MLC	Cell Signaling	Cat # 3671, RRID: AB_330248
Mouse monoclonal anti-Claudin-1	Thermo Fisher Scientific	Cat # 37-490-0, RRID: AB_ 2533323
Mouse monoclonal anti-Occludin	Thermo Fisher Scientific	Cat # 33-150-0, RRID: AB_2533101
Rat monoclonal anti-E-cadherin	Invitrogen	Cat # 13-190-0, RRID: AB_2533005
Rabbit Monoclonal anti-villin antibody	Thermo Fisher Scientific	Cat # MA516408, RRID: AB_2537927

Rabbit polyclonal anti-Caveolin-1 antibody	Santa Cruz Biotechnology	Cat# sc-53564, RRID:AB_628859
Rabbit polyclonal anti-Rab 11 antibody	Thermo Fisher Scientific	Cat# 71-5300, RRID: AB_87868
Mouse Monoclonal Anti-EEA1 Antibody	Santa Cruz Biotechnology	Cat# sc-137130, RRID:AB_2246349
Goat anti-rat IgG (H+L), (Alexa Fluor 555 Conjugate) antibody	Cell Signaling	Cat # 4417, RRID: AB_10696896
Goat anti-mouse IgG (H+L), F(ab')2 Fragment (Alexa Fluor 555 Conjugate) antibody	Cell Signaling	Cat # 4409, RRID: AB_1904022
Goat anti-rabbit IgG (H+L), F(ab')2 Fragment (Alexa Fluor 488 Conjugate) antibody	Cell Signaling	Cat # 4412, RRID: AB_1904025
Goat anti-rabbit ImmPRESS HRP	Vector Labs	Cat # MP-7451, RRID: AB_ 2631198
Goat anti-rat ImmPRESS HRP (M adsorbed)	Vector Labs	Cat # MP-7444, RRID: AB_ 2336530
Isotype control Rabbit IgG	Vector Labs	Cat # I-1000, RRID: AB_ 2336355
Isotype control Rat IgG	Vector Labs	Cat # I-4000, RRID: AB_ 2336356
**Chemicals, Peptides, and Recombinant Proteins**		
Modified Oxford agar Base	Neogen Corporation	Cat # 7428
Modified Oxford agar Base supplement	Neogen Corporation	Cat # 7991
Buffered *Listeria* enrichment broth	Neogen Corporation	Cat # 7675
Buffered *Listeria* enrichment broth supplement	Neogen Corporation	Cat # 7980
DAPI	Cell Signaling	Cat # 4083
FITC-labeled 4 kDa dextran	Sigma-Aldrich	Cat # 46944
ImmPACT DAB (Chromogen)	Vector Labs	Cat # SK-4105
ImmPACT Vector Red (Chromogen)	Vector Labs	Cat # SK-5105
Prolong Gold (Antifade Reagent)	Invitrogen	Cat # P36934
Halt proteases and phosphatase inhibitors	Thermo Fischer Scientific	Cat # PI78443
Amiloride Hydrochloride	Sigma	Cat # PHR1839
Chlorpromazine Hydrochloride	Sigma	Cat # C8138
Pitstop-2	Sigma	Cat # SML1169
Methyl-β-Cyclodextrin	Avanti Polar Lipids	Cat # C4555
β-d-lactosyl-N-octanoyl-l-threo-sphingosine	Sigma	Cat # 860575P
Dynasore	Sigma	Cat # 324410
Bloxall solution (Blocking Solution)	Vector Labs	Cat # SP-6000
WGA-Alexa Fluor 647 conjugate	Thermo Fisher Scientific	Cat # W32466
**Experimental Models: Organisms/Strains**		
Gerbil: Mongolian	Charles River	strain # 243

C57BL/6J Mice	Our Breeding Colony	Jackson Laboratory strain # 000664
Caveolae protein 1 (Cav1) knock-out (Cav-$1^{-/-}$)	Jackson Laboratory	strain # 007083
MLCK knock-out mice lacking the 210-kDa long-chain ($MLCK^{-/-}$) mice	Our Breeding Colony	(7,8)
Cell line: Caco-2	ATCC	Cat # HTB37
Cell line: HCT-8	ATCC	Cat # CCL-244
**Software and Algorithms**		
ImageJ	NIH	https://imagej.nih.gov/ij/
GraphPad Prism 10.0	GraphPad Software	https://www.graphpad.com/scientific-software/prism/
Microsoft Excel 365	Microsoft	https://products.office.com/en-us/excel
Nikon Elements Ver4.60.00	Nikon	https://www.nikoninstruments.com/Products/Software/NIS-Elements-Basic-Research
Zeiss Zen 5	Zeiss	https://www.zeiss.com/microscopy/en/products/software/zeiss-zen.html

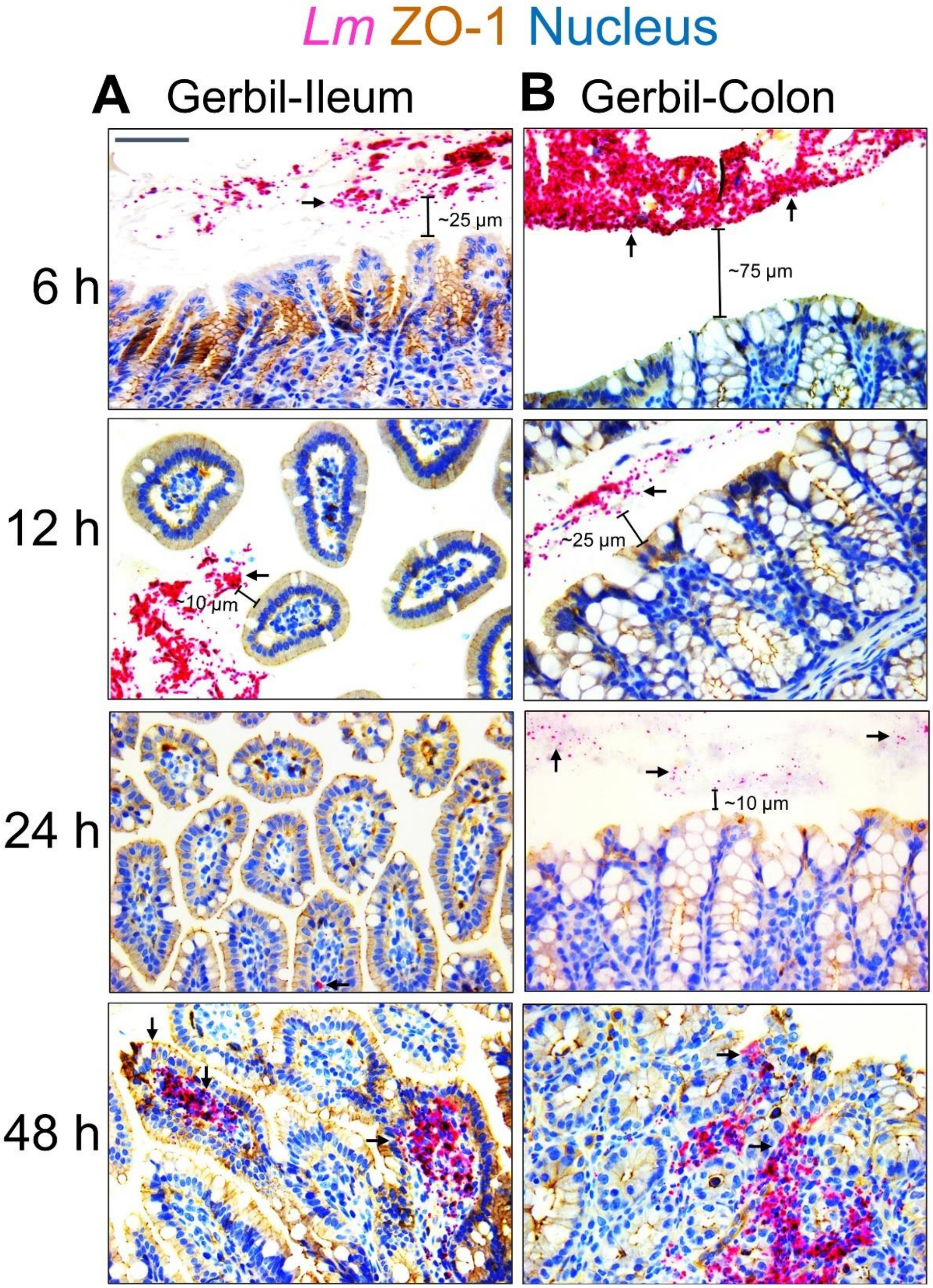

FIG S1. Analysis of intestinal villi invasion of *L. monocytogenes* in orally infected gerbils.

(A-B) Representative zoomed-out micrographs of ileal (**A**) or colonic villi (**B**) dual immunostained for ZO-1 (tight junction, brown) and *Listeria* (red, arrows) and counterstained with hematoxylin to stain the nucleus (blue) from gerbil orally challenged with *Lm* at 6, 12, 24, and 48 hpi; Bars, 50 µm. Translocated *Lm* is observed in the lamina propria (arrows) at 48 hpi but confined in the inner mucus layer in the lumen (arrows) at 6-24 hpi.

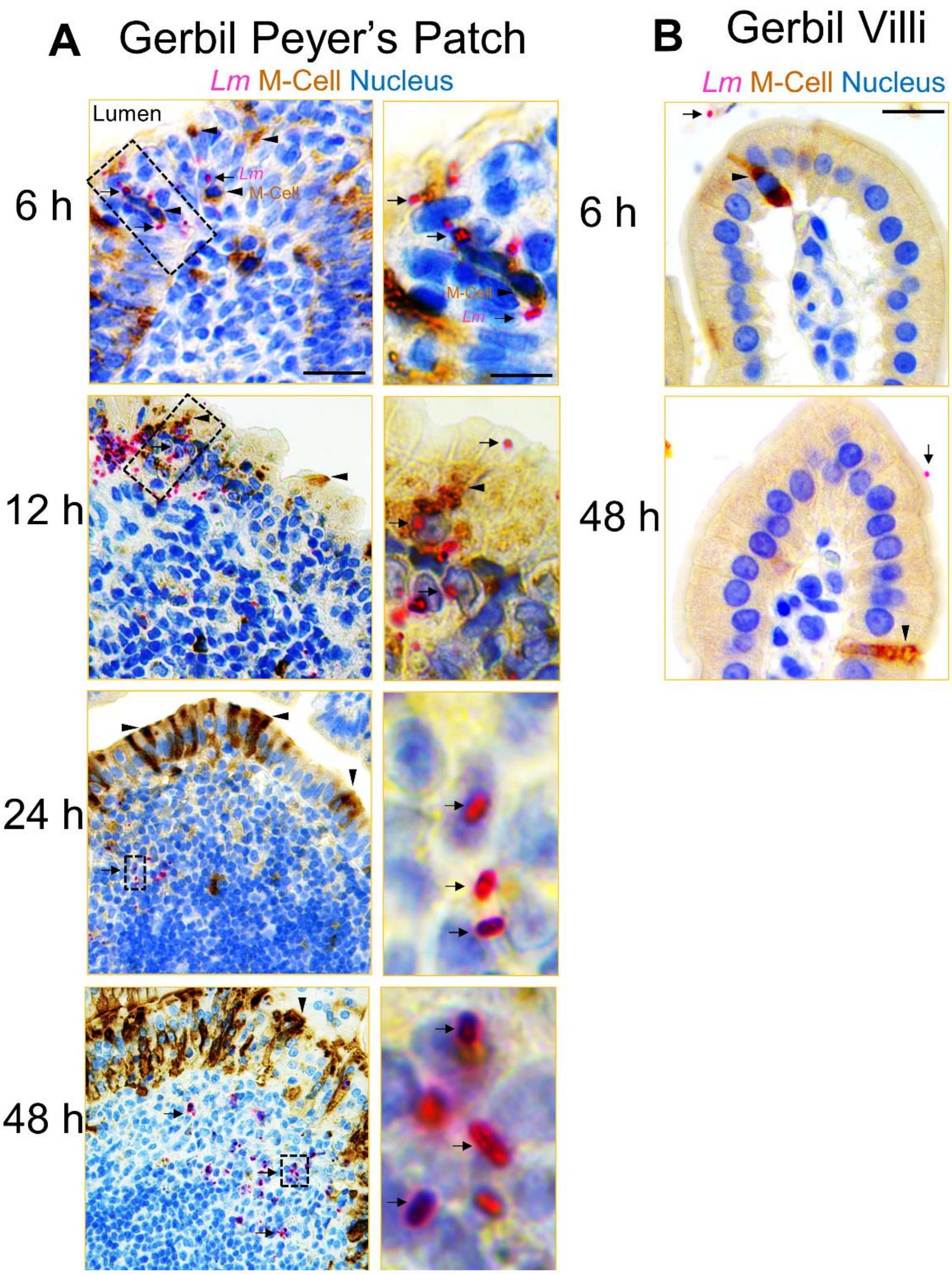

FIG S2. Analysis of M-cell invasion of *L. monocytogenes* in orally infected gerbils.

(A-B) Representative micrographs of gerbil Peyer's patches (PP; **A**) dual immunostained for M-cells (arrowheads; brown) and *Listeria* (red, arrows) and counterstained with hematoxylin to stain the nucleus (blue) from gerbil orally challenged with *Lm* at 6, 12, 24, and 48 hpi; Bars, 50 µm. The boxed areas were enlarged in the right panels; bars, 5 µm. Translocating *Lm* (arrows) across PP M-cells is observed at 6-48 hpi. No association of *Lm* (arrows) with villus M-cell (arrowheads) is observed at 6 or 48 hpi (**B**).

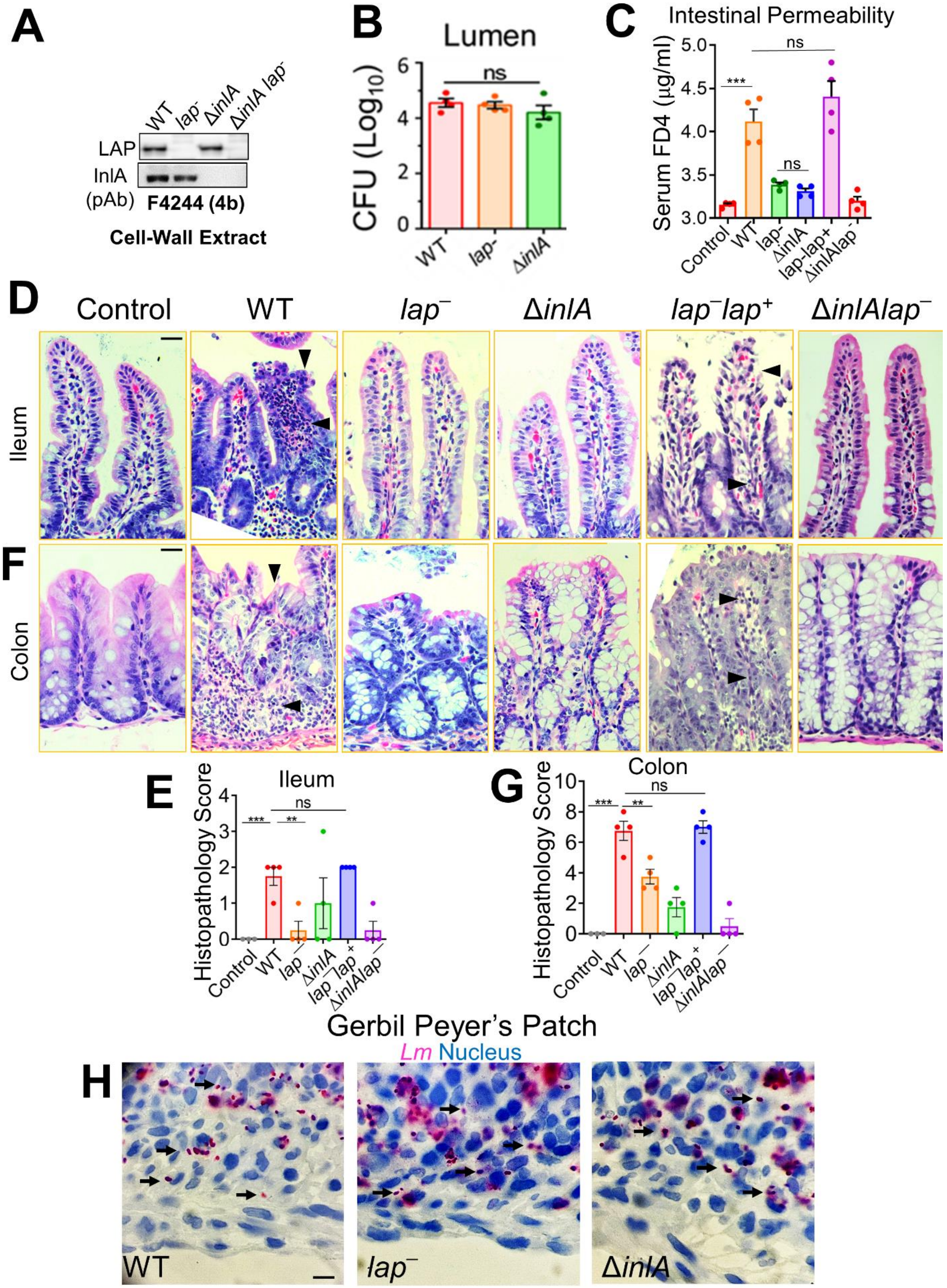

FIG S3. LAP and InlA promote *L. monocytogenes* translocation across the InlA-

permissive intestinal barrier.

(**A**) Immunoblots showing expression of LAP and InlA in whole-cell -extracts of *Lm* WT (F4244) and mutant strain.

(**B**) *Listeria* counts (total CFU) in the intestinal lumen at 48 hpi from gerbil orally challenged with *Lm* WT or mutant strains at 48 hpi; n = 4.

(**C)** Analysis of 4-kDa FITC-dextran (FD4) permeability through the intestinal epithelium of uninfected (control) and *L. monocytogenes*–infected gerbils in serum at 48 h pi. FD4 was administered orally 4-5 h before sacrifice. Data represent mean ± SEM of n = 4 gerbils per treatment from two independent experiments.

(**D-G**) Representative H&E stained picto-micrographs **D** and **F**) and histological score (**E** and **G**) of the ileal (**D** and **E**) and colonic tissues (**E** and **F**) at 48 hpi (bars, 50 µm) from control uninfected gerbils or gerbils orally infected with ~3×10^{8} CFU of *Lm* WT or mutant strains. Arrowheads show increased polymorphonuclear and mononuclear cells infiltrating the base of the villous lamina propria in gerbils challenged with the WT strain; n = 4.

(**H**) Representative micrographs of gerbil Peyer's patches (PP; **C**) immunostained for *Listeria* (red, arrows) and counterstained with hematoxylin to stain the nucleus (blue) from gerbil orally challenged with *Lm* or mutant strains at 48 hpi; Bars, 5 µm.

Each point represents a single gerbil (**B, E, G**). All error bars represent SEM. ***p < 0.001; **p < 0.01; no significance.

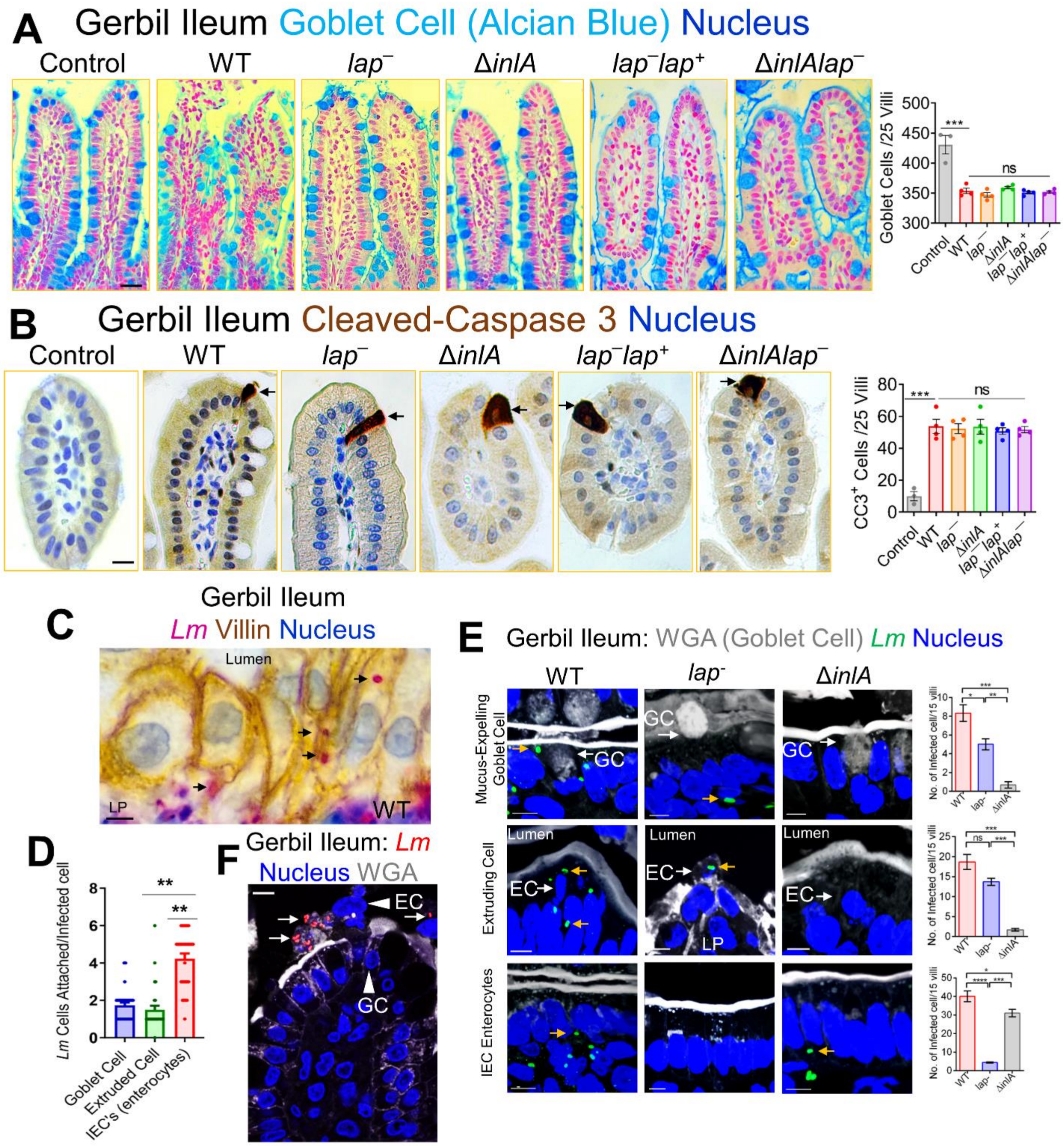

FIG S4: Analysis of *L. monocytogenes* translocation across absorptive enterocytes, goblet, and extruding cells.

(**A-B**) Representative Alcian blue staining (**A**) and Cleaved -Caspase 3 immunostaining (**B**) micrographs of ileal tissue sections from orally challenged with ~3×10^{8} CFU of WT, *lap*⁻ Δ*inlA*, *lap*⁻*lap*⁺ or the Δ*inlA lap*⁻ *Lm*. Quantification of goblet cells (right panel, each point represents an individual gerbil, 4 gerbil per group, n = 100 villi). Scale bar, 25 µm.

(**C**) Representative micrograph micrographs of ileal tissue sections from orally challenged gerbils with ~3×10^{8} CFU of WT *Lm* 48 hpi, dual immunostained for enterocyte-marker villin

(brown) and *Lm* (pink). *Lm* (black arrows) association at and translocation across enterocyte is shown (arrows).

(**D**) Quantification of *Lm* association at and translocation across goblet cells, extruded cells, and enterocytes from immunostained tissue sections orally challenged with ~3×10^8 CFU of WT. n=15 infected cell type from 3 gerbils.

(**E**). Representative confocal immunofluorescence micrographs of intestinal villi section stained for WGA (white), *Lm* (green), and DAPI (blue; nucleus) from *Lm* WT, *lap*⁻ Δ*inlA* infected gerbils at 48 h pi. *Lm* association at and translocation across goblet cell (GC, top panels, yellow arrow) and extruded cell (EC, middle panels, yellow arrow) are InlA-dependent as *ΔinlA* stain shows no association at these sites. *Lm* association (lower panels, yellow arrows) at and translocation across IEC's enterocytes with luminally inaccessible E-cadherin is LAP dependent as *lap*– --strain shows no association at these sites. Graph representing quantitative measurements of *Lm* infected cells (in each cell type or location) of villi images (n = 45 villi) from three gerbils. Each treatment is presented on the right panels.

(**F**) Representative confocal immunofluorescence micrographs of intestinal villi section stained for WGA (white), *Lm* (red, arrows), and DAPI (blue; nucleus) from *Lm* WT infected gerbils at 48 hpi depicting *Lm* association with extruded cells in the intestinal lumen.

Data represent the mean ± SEM, and statistical significance was determined using the one-way ANOVA test followed by Tukey's multiple comparisons. ****p < 0.0001; ***p < 0.001; **p < 0.01; ns, no significance.

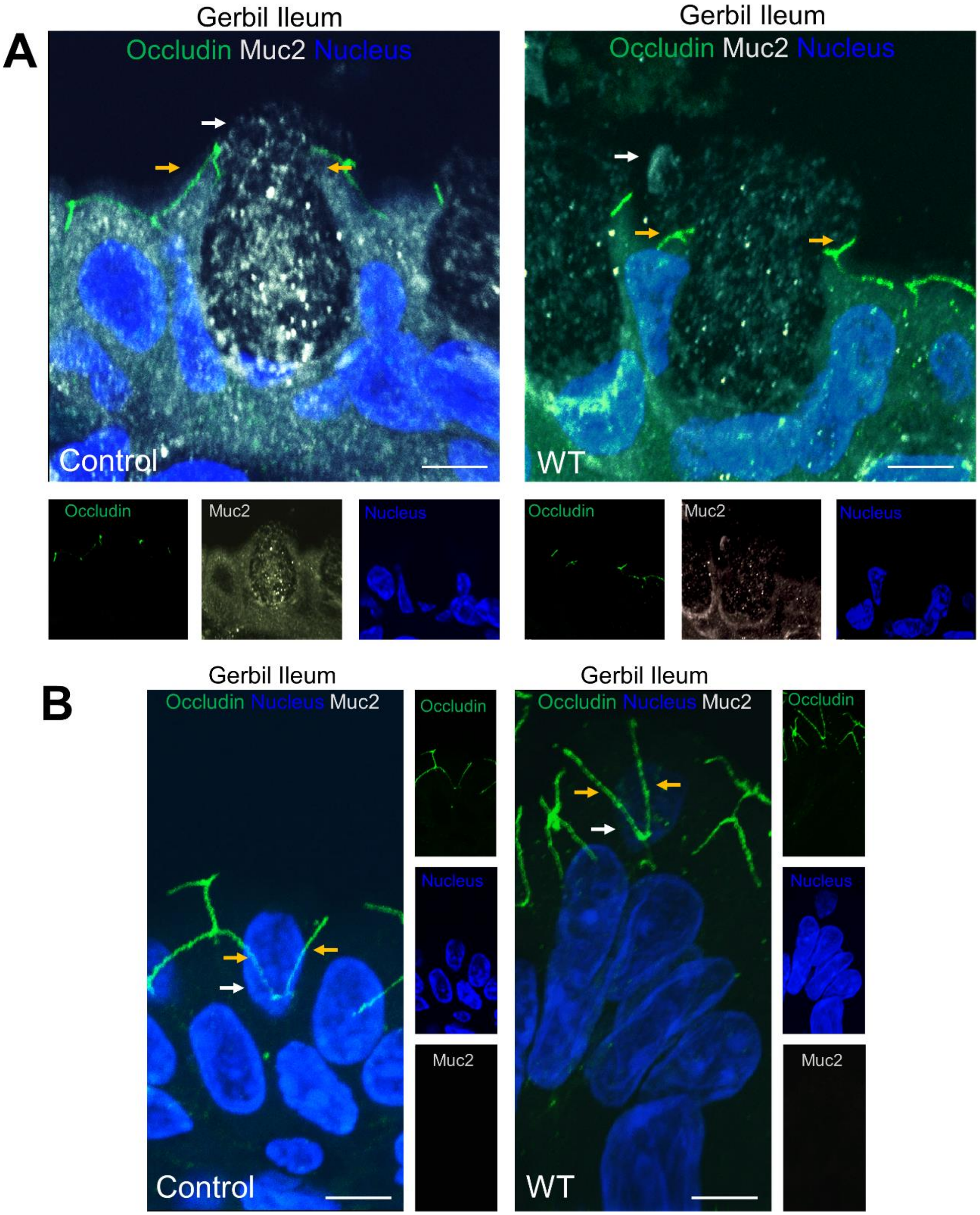

FIG S5: Analysis of occludin localization at goblet cells and extruding cells.

(**A-B**) Representative confocal immunofluorescence picto-micrographs of the gerbil ileal tissue sections immunostained for Occludin or E-cadherin (green), Muc-2 (White, Goblet Cell), and DAPI (blue; nucleus) from uninfected (Control) or WT challenged gerbil at 48 hpi. Bars, 5 µm. A relative redistribution of apical TJ protein occludin is observed in the lateral membranes (yellow arrows) at mucus-secreting (Muc2^{+}) goblet cells (**A**) and the extruding cells (a "V" shape TJ) at the tip of the villi (**B**). Images are representative of five different fields from three gerbils.

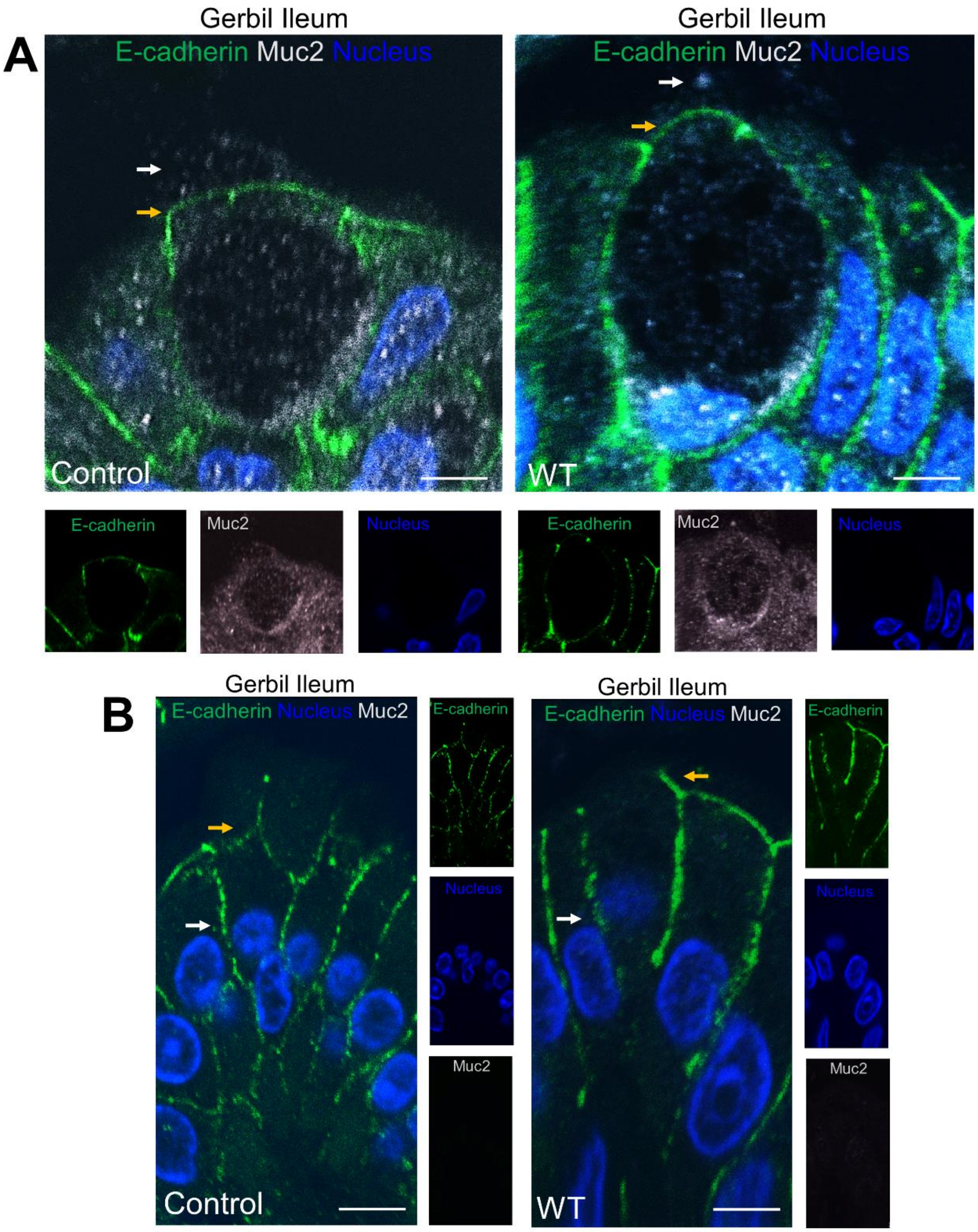

FIG S6: Analysis of E-cadherin localization at mucus-expelling goblet cells and extruding cells.

(**A**). Representative confocal immunofluorescence picto-micrographs of the gerbil ileal tissue sections immunostained for E-cadherin (green), Muc-2 (White, Goblet Cell), and DAPI (blue; nucleus) from uninfected (Control) or WT challenged gerbil at 48 hpi. Bars, 5 µm. Apical enrichment and availability of E-cadherin (yellow arrows) during mucus exocytosis (white arrows) (**A**) and apoptotic cell shedding (**B**, white arrows) at the tip of the intestinal villi. Images are representative of five different fields from three gerbils.

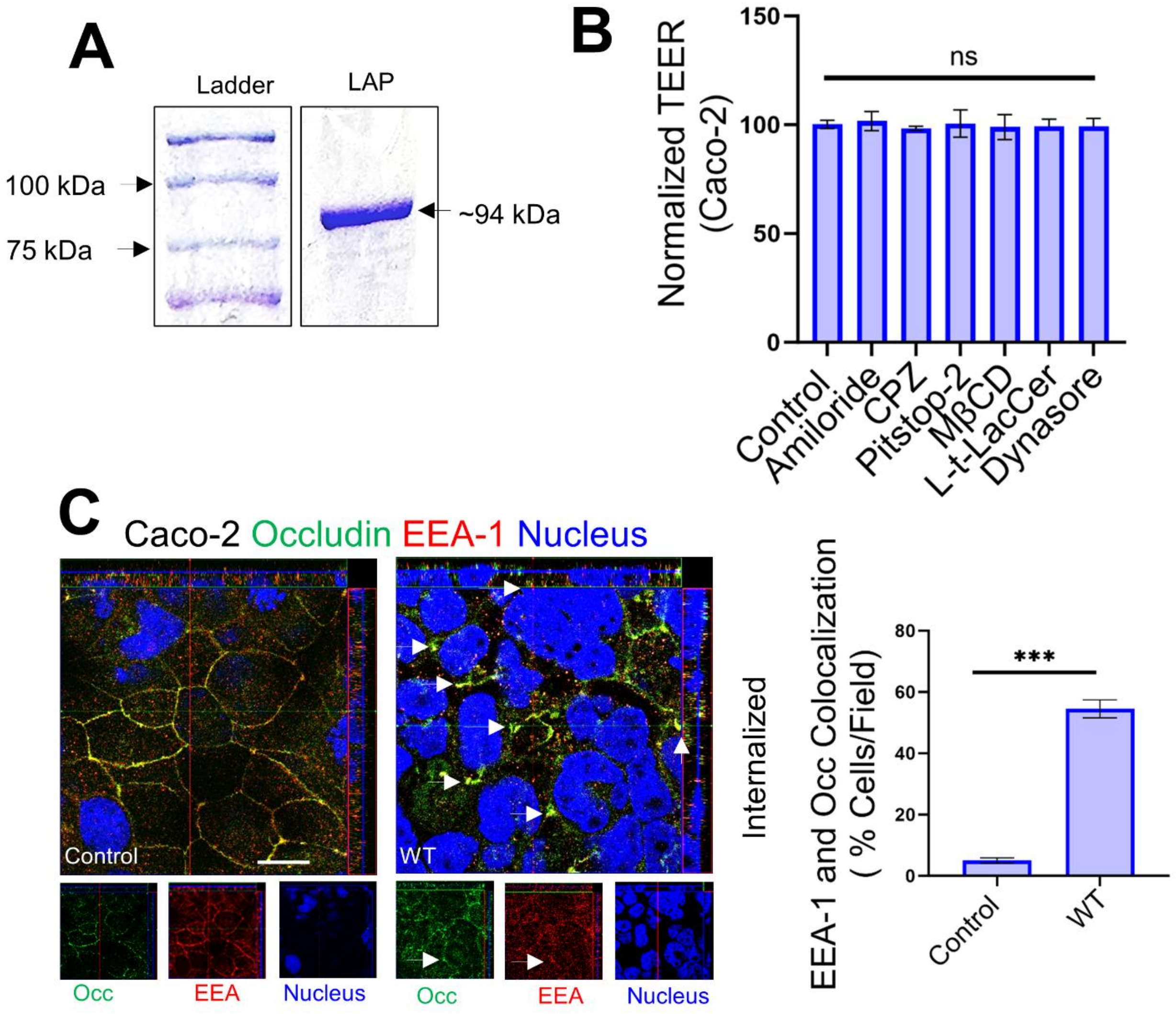

FIG S7. Analysis of LAP-mediated epithelial barrier function.

(**A**) Verification of purity of recombinant LAP by Coomassie blue staining. Well was loaded with 5 µg of purified protein in SDS-PAGE (12.5%- polyacrylamide), electrophoretically separated in gel, and Coomassie stained (left panel)

(**B**) TEER values of filter-insert grown Caco-2 cells before and after treatment with endocytic pathway inhibitors (30 min pre-treatment). Endocytic pathway inhibitors did not affect Caco-2 TEER. The data represent three independent experiments (n=3) and are reported as mean ± SEM.

(**C**) Confocal immunofluorescence micrographs (left) and quantitative analysis (mean ± SEM, n = 6; right) showing colocalization of occludin (green) with EEA-1 in Caco-2 (arrows) following *Lm* WT exposure (MOI; 50, 45 min) Separated channels are shown individually at the bottom of the merged images for clarity.

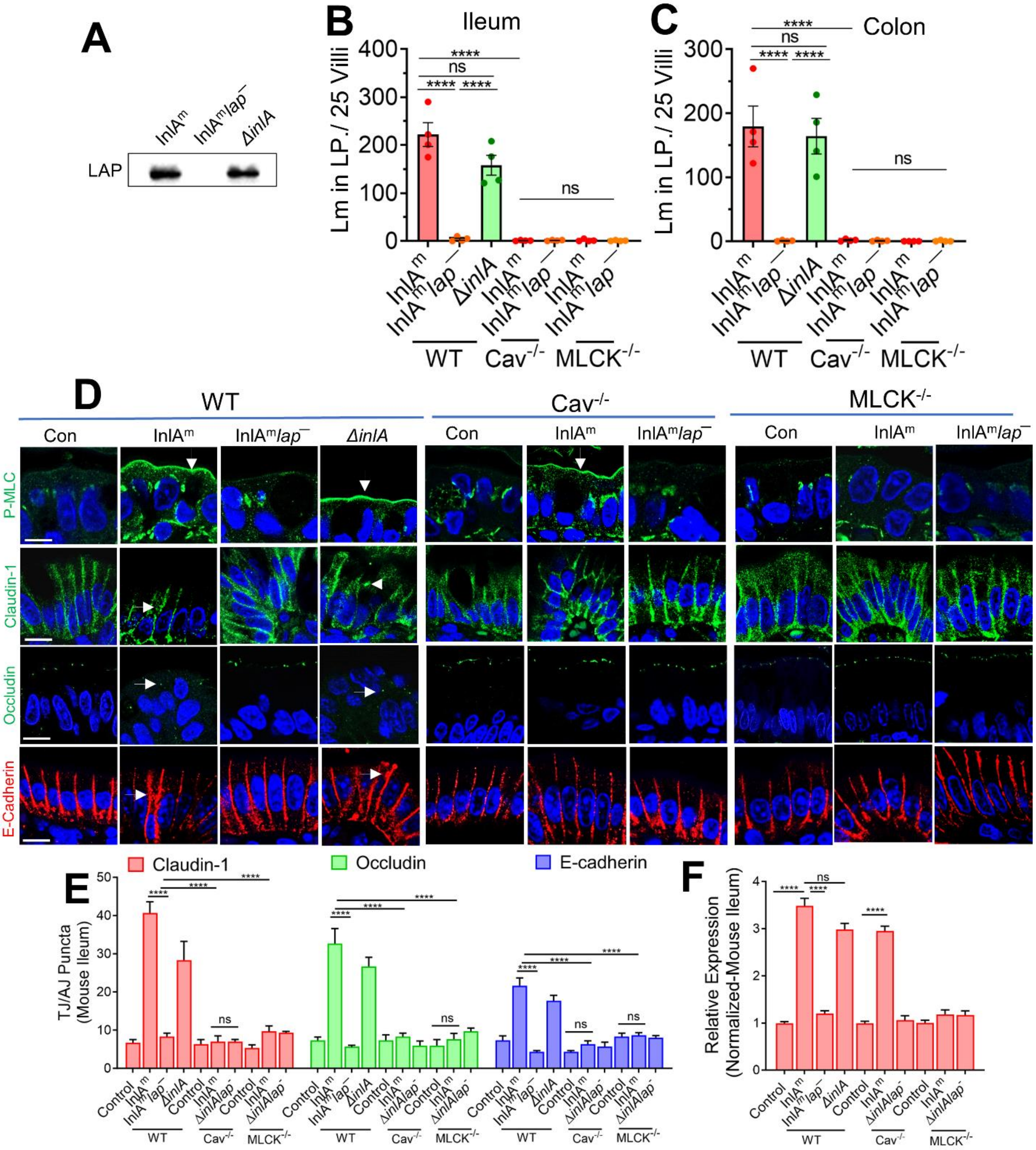

FIG S8. Analysis of *Listeria monocytogenes* (InlA^{m}) translocation and junctional protein expression and distribution in caveolin-1 knockout mice.

(A) Immunoblots showing no expression of LAP in InlA^{m}*lap*$^{–}$ relative to InlA^{m} (F4244), Δ*inlA L. monocytogenes* bacteria.

(**B & C**) Graph representing quantitative measurements of *Lm* counts in the lamina propria from ileal (**B**) or colonic (**C**) villi images (n = 30 villi) from four mice for each treatment.

(**D**) Confocal immunofluorescence micrographs of the ileal tissue sections showing increased expression of P-MLC (green; arrows) and mislocalization (intracellular puncta, endocytosis) of claudin-1, occludin, and E-cadherin (arrows) $InlA^{m}$ challenged WT mice (arrows). Images are representative of five different fields from n=3-4 mice per treatment. Scale bars, 10 μm. LP, Lamina Propria.

(**E& F**) Quantitative analysis (mean ± SEM, n = 3 mice) of P-MLC expression (D) and claudin-1, occludin, and E-cadherin puncta formation (**G**) from images of immunostained ileal tissues orally challenged mice with *Lm* WT or mutant strains at 48 hpi*.*

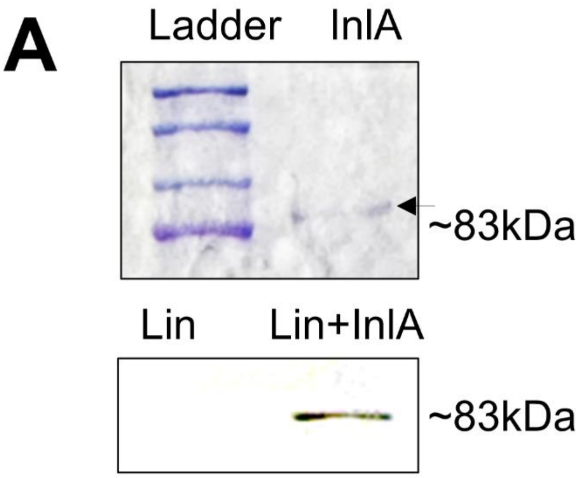

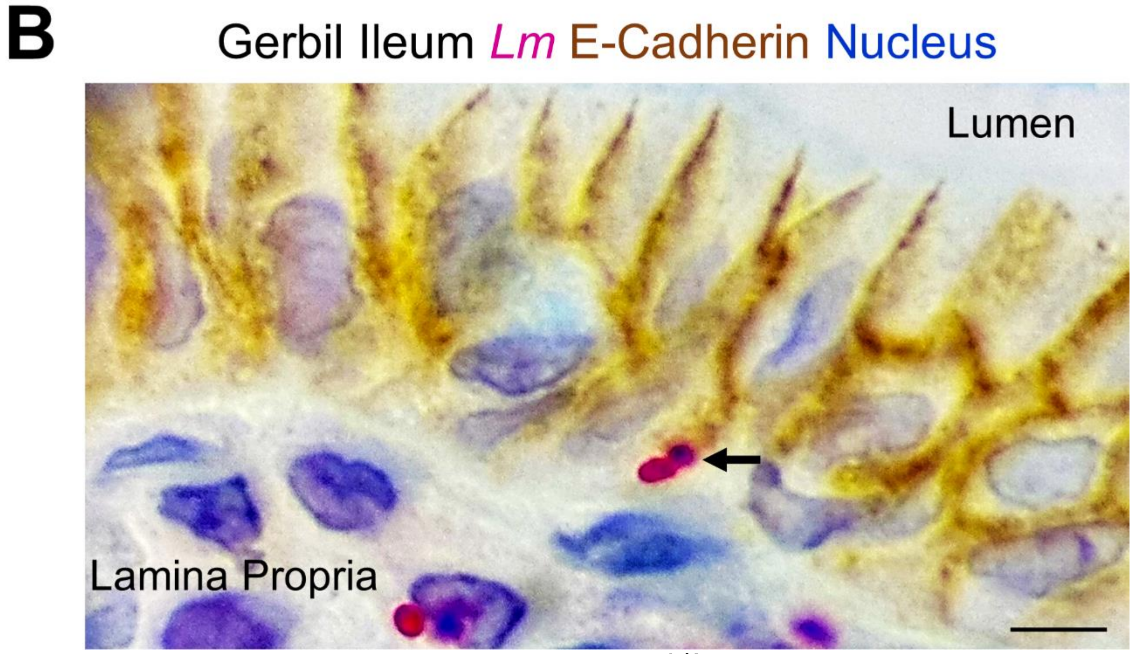

FIG S9. Analysis of the association of InlA on *Lin*^{InlA} and access of the basolateral E-cadherin for *Lm* translocation.

(**A**) Verification of purity recombinant InlA by Coomassie blue staining (top panel). Well was loaded with 2.5 μg of purified protein in SDS-PAGE (12.5%- polyacrylamide), electrophoretically separated in gel, and Coomassie stained (top panel) Immunoblot confirmation of InlA reassociation with Lin (Lin$^{\text{InlA)}}$ (bottom panel).

(B) Representative picto-micrographs of ileal tissue sections immunostained for *Lm* (pink) and E-cadherin (brown) depicting localization of *Lm* at E-cadherin between cell-cell junction, from *L. monocytogenes* WT–infected gerbils at 48 hpi.

REFERENCES

1. **Bailey, T. W., N. C. do Nascimento, and A. K. Bhunia.** 2017. Genome sequence of *Listeria monocytogenes* strain F4244, a 4b serotype. Genome Announcements **5:**e01324-01317.
2. **Jagadeesan, B., O. K. Koo, K. P. Kim, K. M. Burkholder, K. K. Mishra, A. Aroonnual, and A. K. Bhunia.** 2010. LAP, an alcohol acetaldehyde dehydrogenase enzyme in *Listeria* promotes bacterial adhesion to enterocyte-like Caco-2 cells only in pathogenic species. Microbiology **156:**2782-2795.
3. **Burkholder, K. M., and A. K. Bhunia.** 2010. *Listeria monocytogenes* uses *Listeria* adhesion protein (LAP) to promote bacterial transepithelial translocation, and induces expression of LAP receptor Hsp60. Infect. Immun. **78:**5062-5073.
4. **Bai, X., D. Liu, L. Xu, S. Tenguria, R. Drolia, N. L. F. Gallina, A. D. Cox, O.-K. Koo, and A. K. Bhunia.** 2021. Biofilm-isolated *Listeria monocytogenes* exhibits reduced systemic dissemination at the early (12–24 h) stage of infection in a mouse model. npj Biofilms and Microbiomes **7:**1-16.
5. **Geng, T., M. T. Morgan, and A. K. Bhunia.** 2004. Detection of low levels of *Listeria monocytogenes* cells by using a fiber-optic immunosensor. Appl. Environ. Microbiol. **70:**6138-6146.
6. **Mendonca, M., N. Conrad, F. Conceicao, A. Moreira, W. da Silva, J. Aleixo, and A. Bhunia.** 2012. Highly specific fiber optic immunosensor coupled with immunomagnetic separation for detection of low levels of *Listeria monocytogenes* and *L. ivanovii*. BMC Microbiol. **12:**275.
7. **Clayburgh, D. R., T. A. Barrett, Y. Tang, J. B. Meddings, L. J. Van Eldik, D. M. Watterson, L. L. Clarke, R. J. Mrsny, and J. R. Turner.** 2005. Epithelial myosin light chain kinase–dependent barrier dysfunction mediates T cell activation–induced diarrhea in vivo. J. Clin. Invest. **115:**2702-2715.
8. **Drolia, R., S. Tenguria, A. C. Durkes, J. R. Turner, and A. K. Bhunia.** 2018. *Listeria* adhesion protein induces intestinal epithelial barrier dysfunction for bacterial translocation. Cell Host & Microbe **23:**470-484.
